# Metabolomics insights into Charcot–Marie–Tooth disease: toward biomarker discovery

**DOI:** 10.3389/fneur.2025.1543547

**Published:** 2025-05-19

**Authors:** Signe Setlere, Theresa Schiemer, Annija Vaska, Linda Gailite, Dmitrijs Rots, Viktorija Kenina, Kristaps Klavins

**Affiliations:** ^1^Department of Neurology and Neurosurgery, Children’s Clinical University Hospital, Riga, Latvia; ^2^Department of Doctoral Studies, Riga Stradins University, Riga, Latvia; ^3^Faculty of Natural Sciences and Technology, Institute of Biomaterials and Bioengineering, Riga Technical University, Riga, Latvia; ^4^Baltic Biomaterials Centre of Excellence, Headquarters at Riga Technical University, Riga, Latvia; ^5^Institute of Oncology and Molecular Genetics, Riga Stradins University, Riga, Latvia; ^6^Genetics Laboratory, Children’s Clinical Univeristy Hospital, Riga, Latvia; ^7^Department of Biology and Microbiology, Riga Stradins University, Riga, Latvia; ^8^Rare Neurological Disease Centre, Pauls Stradins Clinical University Hospital, Riga, Latvia

**Keywords:** polyneuropathy, genetic and inherited disorders, metabolome, Charcot–Marie Tooth disease, biomarker

## Abstract

**Introduction:**

Charcot–Marie–Tooth disease (CMT) is a group of rare neuropathies but still the most common hereditary neuromuscular disorder with heterogeneous phenotype and usually slow progression. Currently, there are no approved treatments or validated biomarkers for sensitive monitoring of disease progression.

**Objectives:**

This study aimed to analyse selected plasma metabolite concentrations in a CMT cohort and compare them to healthy controls. For this purpose, 84 patients and 34 controls were enrolled in the study.

**Results:**

We detected a total of 33 metabolites from which acetylcarnitine was found elevated and glycine was found decreased in CMT patients. In addition, the CMTX1 subgroup has decreased valine levels compared to controls. However, further analysis revealed poor disease predictive abilities of the detected metabolites for any CMT group. Furthermore, we found no associations of these metabolites with CMT severity.

**Conclusion:**

Our study data provide information about plasma metabolite levels in CMT patients. However, these findings suggest that the metabolites mentioned above might be unspecific biomarkers of neuropathy and do not reflect disease severity.

## Introduction

Charcot–Marie–Tooth (CMT) disease is a clinically and genetically heterogeneous group of disorders with the phenotype of usually slowly progressive, chronic neuropathy affecting both the motor and the sensory nerves. CMT disease is the most common hereditary neuromuscular disorder, with an estimated prevalence of 1/2500 (1). It presents with progressive distal muscle atrophy and weakness, distal sensory loss, and foot deformities that can seriously reduce a patient’s quality of life. Currently, there are no approved therapies, however, promising new treatments are approaching the clinical translation stage (2). Therefore, given the slow progressive nature of the disease, sensitive disease progression and treatment responsive biomarkers become crucial for the upcoming clinical trials.

In the recent years, metabolome analysis has been widely applied for discovering diagnostic and prognostic markers as well as for uncovering underlying pathophysiological mechanisms of diseases, including polyneuropathies. Metabolomics is the characterization of small molecules (<1,500 Daltons) in biological matrices using analytical chemistry techniques (3). Knowledge about metabolites involved in different metabolic pathways that are affected in polyneuropathies can be helpful to provide insight into disease mechanisms, and potentially identify biomarkers and therapeutic targets. Numerous studies have confirmed that some plasma metabolites are associated with diabetic neuropathy (4–9). Soldevilla et al. (10) used untargeted metabolomic approach of plasma samples in a cohort of 42 CMT1A patients and 15 controls. They identified 12 plasma metabolites that might be promising candidates for CMT1A disease biomarkers. However, there are no widely accepted metabolite biomarkers for CMT, and data about metabolic profiles in hereditary neuropathies are still lacking.

In this study, we analysed selected metabolites and metabolite ratios in CMT patient plasma and compared them age- and sex-matched healthy controls. Further, we evaluated serum metabolites in patients with CMT1A, CMTX1, CMT2A, HINT1, other genetic subtypes, unknown genetic type and mild, medium, severe degree of CMT. Moreover, we aimed to investigate the association between CMT severity and potential biomarkers in plasma.

## Methods

### Patient’s evaluation and blood sampling

A cohort of 84 patients form geneticists’, neurologists’ and paediatric neurologists’ clinical practices with CMT and 34 healthy controls were recruited to this study as described before (11, 12). Twelve CMT patients and 5 healthy controls were under 18 years of age. All CMT individuals underwent genetic testing including *PMP22* duplication/deletion analysis, and exome sequencing with analysis of hereditary neuropathy-associated genes, as described before (11). CMT disease severity was evaluated by an expert neurologist with widely accepted CMT Neuropathy Score Version 2 (CMTNSv2). In this study, patients were divided based on the severity into three groups: mild (CMTNSv2 score 0 to 10), medium (CMTNSv2 score 11 to 20), and severe (CMTNSv2 score >21) group (13).

As a control group, our study included age- and sex-matched healthy individuals without known neurological diseases or symptoms.

Blood sampling and storage were conducted following a strict standard operating procedure. Briefly, blood samples from patients and controls were taken in an outpatient setting by certified medical staff and processed within 1 h. Blood was collected into EDTA-containing tubes and centrifuged at 20°C at 3,500 rpm for 10 min. Plasma was then aliquoted and stored at −20°C.

### Metabolite analysis

Targeted plasma metabolic analysis was performed by ultrahigh performance liquid chromatography-mass spectrometry (UHPLC–MS) to determine plasma levels of 55 selected metabolites, from which 33 were detected (Supplementary Table 1) in plasma samples from CMT patients and healthy controls. These metabolites were selected as they are routinely screened via mass spectrometry (MS/MS) in clinical laboratories during newborn screening (14). By measuring these established markers, changes detected in their concentrations in CMT patients’ blood could offer a readily implementable diagnostic tool for this disorder.

The LC–MS analysis was performed on a Dionex 3,000 HPLC system (Thermo Scientific) coupled with an Orbitrap Q Exactive (Thermo Scientific) mass spectrometer. An ACQUITY UPLC BEH Amide, 1.7 μm, 2.1×100 mm analytical column (Waters) equipped with a VanGuard: BEH C18, 2.1×5 mm pre-column (Waters) was used for chromatographic separation. The column temperature was 40°C; the sample injection volume was 2 μl. Mobile phase A—0.15% formic acid (v/v) and 10 mM ammonium formate in water was used, and as mobile phase B—0.15% formic acid (v/v) in 85% acetonitrile (v/v) with 10 mM ammonium formate was used. The gradient elution with a flow rate of 0.4 ml/min was performed resulting in a total analysis time of 17 min. The Orbitrap Q Exactive (Thermo Scientific) mass spectrometer was operated in a positive electrospray ionization mode. The following parameters were used for the ion source: spray voltage 3.5 kV, aux gas heater temperature 400°C, capillary temperature 350°C, aux gas flow rate 12, and sheat gas flow rate 50. The MS detection was performed in a full MS scan mode; the scan range was set to m/z 50 to 400, mass resolution 35,000, AGC target 1e6, maximum IT 50 ms. The Trace Finder 4.1 software (Thermo Scientific) was used for data processing. A seven-point linear calibration curve with internal standardization and 1/x weighing was constructed to quantify of the metabolites.

### Statistical analysis

Clinical data distribution was expressed as medians with interquartile ranges (IQRs). Statistical analysis was performed with Prism 9 and MetaboAnalyst 6.0.^1^

Each metabolite concentration was normalized to the sample medium to minimize the effect of different measurement batches. By default, we excluded metabolites with >20% missing values, which led to no exclusions in our dataset. Fold change and *p*-value were plotted as volcano plots using FC > 1.3 and *p*-value < 0.05 as significance cut-offs (Prism9). Normalized concentrations were plotted as violin plots, and significance testing was done using Sidak’s multiple comparison test. Orthogonal partial least squares-discriminant analysis (OPLS-DA) was performed using MetaboAnalyst 6.0. For this, data was log10 transformed and scaled by mean centering and dividing by the standard deviations square root of each metabolite.

### Standard protocol approval and patient consent

The study was approved by the Central Medical Ethics Committee of Latvia (No. 3/18-03-21). Written informed consent was obtained from all participants in the study.

## Results

This study included 84 CMT patients and 34 healthy controls. The patient group was subdivided according to the genetic findings: CMT1A (*n* = 37), CMTX1 (*n* = 17), CMT2A (*n* = 4), HINT1 (*n* = 5), other genetic subtypes (*n* = 14), unknown genetic type (*n* = 7). There was no significant difference in sex (chi-square, χ^2^ = 0.345, *p* = 0.557) or age (independent samples *t*-test, *t* = 0.143, *p* = 0.509) between CMT and control groups (Table 1).

**Table 1:** CMT study groups.

Study participants	Number of patients (male/female)	Mean age (SD)
All CMT patients	84 (37/47)	39 (17)
CMT1A (*PMP22* dup)	37 (13/22)	38 (17.3)
CMTX1 (*GJB1*)	17 (6/8)	35.8 (16.4)
CMT2A (*MFN2*)	4 (2/2)	34 (17.7)
CMT2N (*AARS1*)	4 (2/2)	38.1 (19)
NMAN (*HINT1*)	5 (2/3)	44.1 (20.7)
CMT2F (*HSPB1*)	2 (1/1)	39.5 (18)
HMN5C (*BSCL2*)	3 (1/2)	38.8 (17.8)
CMT2Z (*MORC2*)	1 (1/0)	48
SMALED2A (*BICD2*)	1 (0/1)	50
CMT1B (MPZ)	1 (0/1)	65
HSP (SPG11)	1 (1/0)	21
CMT with unknown monogenic cause	7 (3/4)	39.2 (18.2)
Control group	34 17/17	39.5 (19.7)

**Figure 1:**
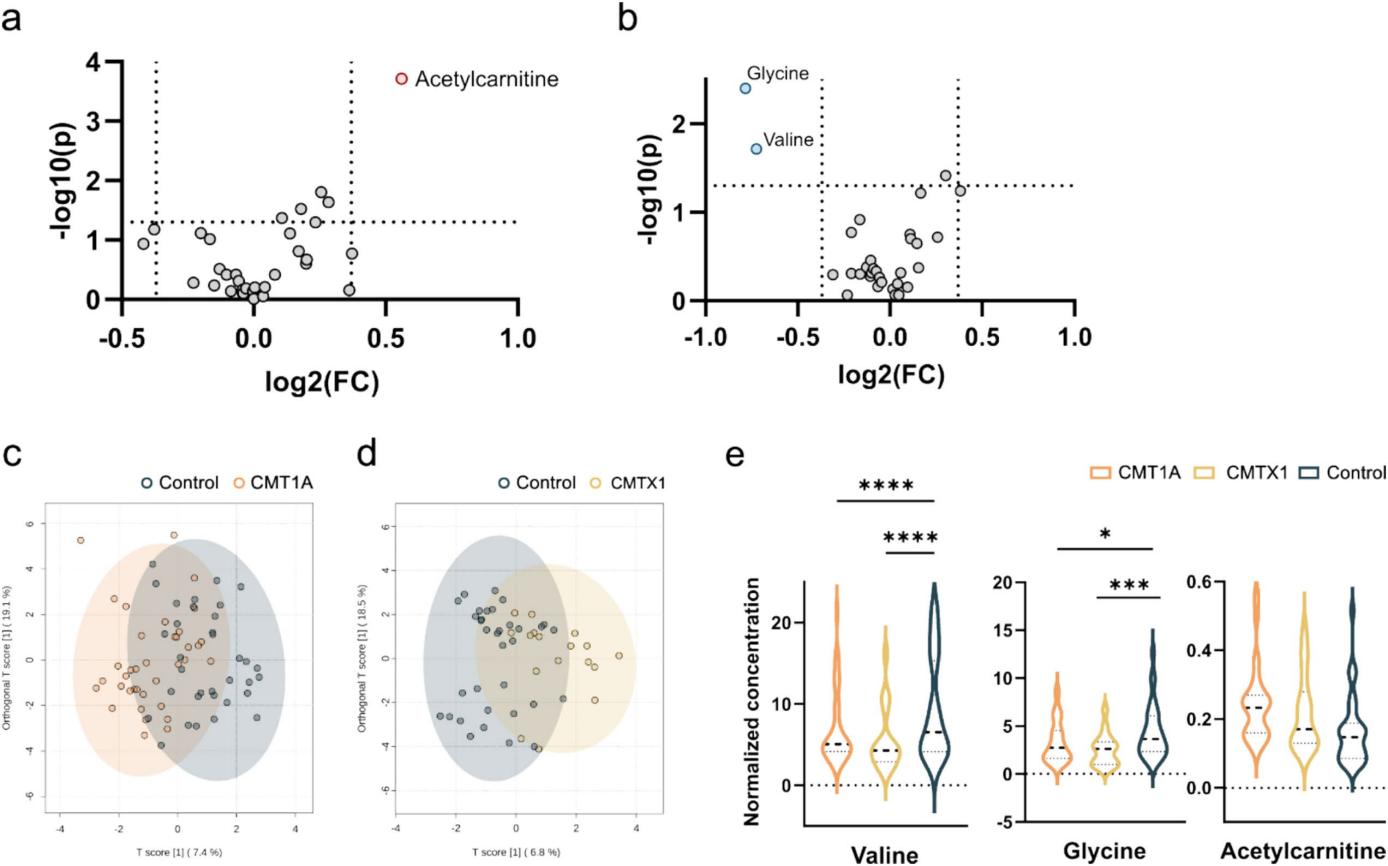
Visualization of differential metabolite profiles compared to healthy controls of **(a)** CMT1A and **(b)** CMTX1 using V-plots. Significance thresholds are indicated with dashed lines (FC > 1.3, *p* < 0.05). oPLSDA plots of **(c)** CMT1A and **(d)** CMTX1. **(e)** Violin plots of metabolites identified to be significantly chqnged in the volcano plots (**** *p* < 0.0001, ** *p* < 0.01, *p < 0.05).

A total of 33 metabolites (Supplementary Table 1) were analysed in plasma. We used univariate statistical analysis to screen for differential plasma metabolites between separate genetic CMT groups with a sufficient number of cases [CMT1A (*n* = 37) and CMTX1 (*n* = 17)] and the control group. We identified differential metabolites with the volcano plot (V-plot) and OPLS-DA (Figures 1a–d). We found that acetylcarnitine in the CMT1A group and glycine and valine in the CMT1X group are different from the controls at level *p* < 0.05, VIP > 1, and FC > 1.3 (Figure 1e, Tables 2 and 3).

**Table 2:** Differential plasma metabolites between CMT1A patients and healthy controls.

Metabolite	*p*-value	VIP	FC	Control group (median [IQR])	CMT1A (median [IQR])
L-acetylcarnitine	1.9384E-4	2.4906	1.4742	10.8350 [9.86]	12.1200 [7.75]

Differential metabolites were selected according to VIP > 1, FC > 1.3, and *p* < 0.05. Values are expressed as medians [IQR]. *p* values were calculated from a *t*-test for continuous variables; VIP, variable influence on projection; CMT, Charcot–Marie–Tooth disease.

**Table 3:** Differential plasma metabolites between CMTX1 patients and healthy controls.

Metabolite	*p*-value	VIP	FC	Control group (median [IQR])	CMTX1 (median [IQR])
Glycine	0.0039756	2.2636	0.58076	299.9250 [154.25]	125.3300 [241.10]
L-valine	0.019376	1.3453	0.60479	529.5950 [455.44]	323.2700 [317.20]

Differential metabolites were selected according to VIP > 1, FC > 1.3, and *p* < 0.05. Values are expressed as medians [IQR]. *p* values were calculated from the *t*-test for continuous variables; VIP, variable influence on projection; CMT, Charcot–Marie–Tooth disease.

Further we screened for differential metabolites in all CMT patients and controls.

**Figure 2:**
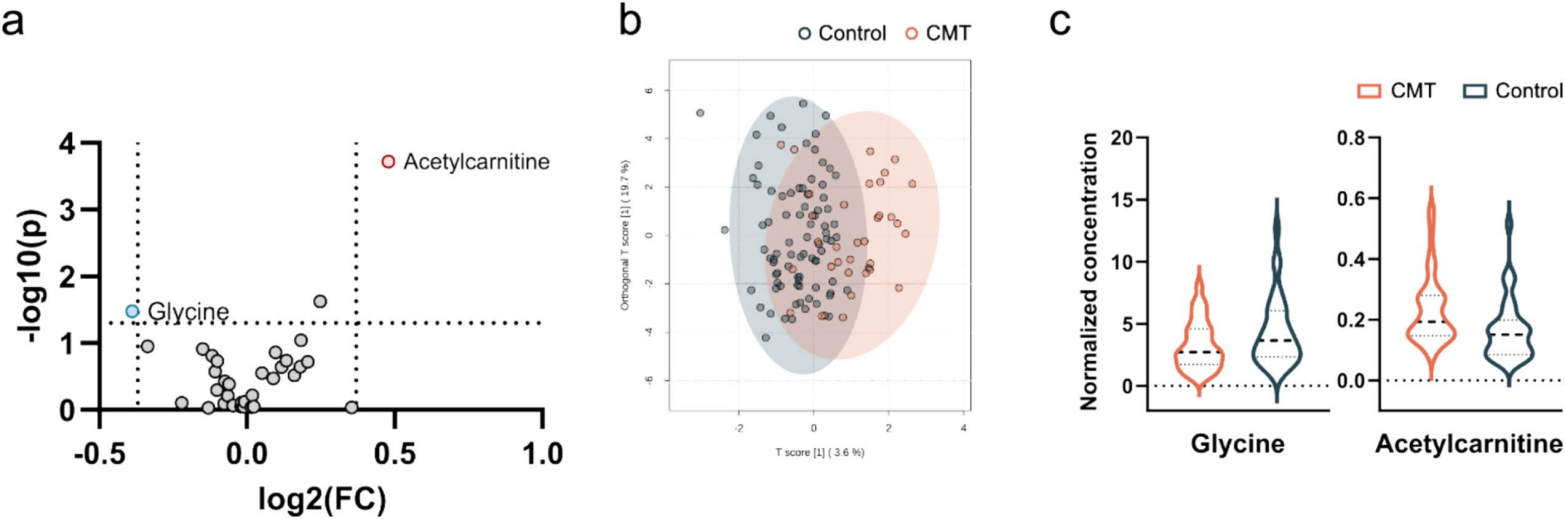
Visualization of differential metabolite profiles in all CMT cases compared to healthy controls using **(a)** V-plot. Significance thresholds are indicated with dashed lines (FC > 1.3, *p* < 0.05). **(b)** OPLS-DA score plot of CMT versus control. **(c)** Violin plot of glycine and acetylcarnitine as the significant metabolites identified in the V-plot (*p* < 0.05).

We identified two plasma metabolites that were significantly changed to controls without being able to separate CMT subtypes based on their metabolic profiles (Figures 2a,b). The plasma ratio of acetylcarnitine was elevated and the plasma ratio of glycine was decreased in the CMT compared with controls (Mann–Whitney U test, U = 1773.000, *p* = 0.04; U = 1018.000, *p* = 0.15, respectively) (Figure 2c, Table 4).

**Table 4:** Differential plasma metabolites between CMT patients and healthy controls.

Metabolite	*p*-value	VIP	FC	Control group (median [IQR])	CMT (median [IQR])
L-acetylcarnitine	2.7234E-4	2.232	1.3883	10.8350 [9.86]	13.4850 [7.49]
Glycine	0.03318	1.5599	0.76581	299.9250 [154.25]	194.5500 [224.53]

Differential metabolites were selected according to VIP > 1, FC > 1.3, and *p* < 0.05. Values are expressed as medians [IQR]. *p* values were calculated from the *t*-test for continuous variables; VIP, variable influence on projection; CMT, Charcot–Marie–Tooth disease.

### Predictive abilities of CMT-related biomarkers

**Figure 3:**
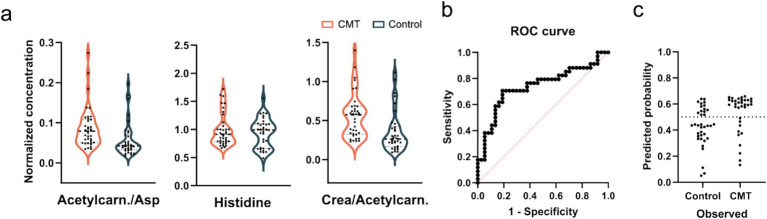
**(a)** Violin plots of one metabolite and two ratios were used for the CMT1A predictive model. Concentrations were normalized to the average of each sample. ANOVA with Bonferroni correction shows no significant differences. **(b)** ROC curves of a predictive model based on multiple linear regression and **(c)** classifications of samples using the constructed model.

Next, we attempted to classify CMT1A and CMTX1 patients, as well as all CMT patients with the measured metabolite levels and their ratios using machine learning, which all achieved an AUC < 0.74 (Supplementary Figure 1). As the most promising results we took a closer look at the important features of the CMT1A model (Figure 3), which was the most significant CMT subgroup, and constructed a classification based on multiple regression of three features. The ROC curve (Figure 3b) achieved an AUC of 0.73 and sample classification (Figure 3c) indicates a poor separation ability.

### Metabolic profiles in CMT of different severity

Further, we divided the 66 patient who had their CMTNSv2 severity score measured into three severity groups: mild (CMTNSv2 score 0–10; *n* = 34), moderate (CMTNSv2 score 11–20; *n* = 27), and severe (CMTNSv2 score > = 21; *n* = 5) groups. Pairwise PCA and OPLS-DA analysis identified two differential metabolites (*p* < 0.05; VIP > 1; FC > 1) in moderate versus severe (Table 5) and one metabolite in mild versus severe CMT group comparison (Table 6).

**Table 5:** Differential plasma metabolites between moderate and severe CMT patients.

Metabolite	*p*-value	VIP	FC	Moderate CMT (median [IQR])	Severe CMT (median [IQR])
L-tyrosine	0.023081	1.8717	0.74866	80.7300 [64.82]	77.1100 [88.74]
L-acetylcarnitine	0.033947	2.09858	0.67648	12.6300 [6.73]	16.1300 [16.80]

Differential metabolite ratios were selected according to VIP > 1, FC > 1.3, and *p* < 0.05. Values are expressed as medians [IQR]. *p* values were calculated from a *t*-test for continuous variables; VIP, variable influence on projection; CMT, Charcot–Marie–Tooth disease.

**Table 6:** Differential plasma metabolites between mild and severe CMT patients.

Metabolite	*p*-value	VIP	FC	Mild CMT (median [IQR])	Severe CMT (median [IQR])
L-proline	0.024678	1.3111	0.73151	291.2650 [196.39]	311.5200 [142.87]

Differential metabolite ratios were selected according to VIP > 1, FC > 1.3, and *p* < 0.05. Values are expressed as medians [IQR]. *p* values were calculated from the *t*-test for continuous variables; VIP, variable influence on projection; CMT, Charcot–Marie–Tooth disease.

However, we could not find significant changes in tyrosine, acetylcarnitine or proline between the severity groups or with controls. In addition, correlation analysis showed no metabolites with a score >0.5, indicating a poor correlation of our measured metabolites (Supplementary Figure 2).

## Discussion

CTM disease biomarkers for treatment response prediction are a bottleneck for finding a specific treatment approach. In this study, we searched for plasma metabolites in a large CMT cohort (Table 1) to improve understanding of the molecular basis of the disease. Moreover, we aimed to identify future biomarkers of the disease.

In the present study, we analysed 33 metabolites in plasma. We identified 2 metabolites (acetylcarnitine and glycine) that changed most significantly between CMT patients and controls (Figure 2). Acetylcarnitine levels in the CMT cohort were higher than in the controls (Table 4). In general, measurements of the carnitine pool have been used to identify the disease and predict mortality among disorders such as diabetes, sepsis, cancer, and heart failure (15). Acetylcarnitine is short-chain acylcarnitine, with plasma levels reaching nearly 80% of all acylcarnitines (16–19). Acylcarnitines are recognized for facilitating fatty acid β-oxidation (FAO) in mitochondria and peroxisomes, producing energy to sustain cell activity (19, 20). Blood concentrations of acylcarnitine reflect intracellular levels and the regulation of acetyl-CoA and free CoA via carnitine acetyl-CoA transferase (19, 21). Increased production of acylcarnitine represents a critical mechanism to buffer the metabolic status between fed (glucose oxidation) and fasted (fat oxidation) states, referred to as metabolic flexibility (22, 23). Therefore, persistent elevations in blood concentrations of acylcarnitine over time may represent a signal of metabolic inflexibility (15). Previous research in mouse models demonstrates that primary insulin resistance destroys insulin signalling transduction in Schwann cells, depleting important myelin lipid components and eventually leading to demyelination (24). In addition, disruption of Schwann cells mitochondria leads to transition from synthesis to oxidation of fatty acids and secondary acylcarnitine formation and accumulation (24–26). Poorabbas et al. (27) showed that Type II diabetes patients with complications (e.g., neuropathy) had 25% lower serum L-carnitine levels than diabetic patients without complications that could be explained by increased production of other acylcarnitines. Zhenni et al. (25) proved that elevated plasma acetylcarnitine levels are positively associated with diabetic polyneuropathy risk. To sum up, acetylcarnitine plays a role in diabetic neuropathy pathophysiology and plasma acetylcarnitine levels correlate with diabetic polyneuropathy development. So far elevated plasma acylcarnitine levels in hereditary neuropathy have been reported before in CMT caused by a variant in the *HADHB* gene encoding the β-subunit of mitochondrial trifunctional protein (28). These findings suggest that acetylcarnitine might be an unspecific metabolite marker in neuropathy.

Further, we analysed plasma metabolites between genetic groups. In CMT1A group acetylcarnitine level was elevated compared to the control group (Figure 1, Table 2). In the CMTX1 group glycine and L-valine concentration in serum was decreased compared to the control group (Figure 1, Table 3). Low systemic glycine is emerging as a hallmark of peripheral nerve disorders, correlating with peripheral neuropathy (29). In mice models, a reduction in serine and glycine levels in plasma after dietary restriction is sufficient to increase plasma and tissue levels of deoxysphingolipids and causes functional peripheral sensory deficits (30). Moreover, it is reported that valine, leucine and isoleucine biosynthesis is one of the main pathways involved in diabetic polyneuropathy (8). Although glycine and valine metabolism contribute to the pathogenesis of peripheral neuropathy, the exact mechanism of involvement is not fully understood. In our study valine was decreased only in one genetic group. Therefore, it remains unclear whether these metabolites could potentially become specific CMT biomarkers.

Next, we aimed to explore the predictive abilities of CMT-related metabolite markers (Supplementary Figure 1). Soldevilla et al. (10) found that from 12 differential metabolites in the CMT1A cohort, four of them (glutaminyl-serine, sphingosine-1-phosphate, tryptophan and leucine) could provide potential biomarkers of the disease as assessed by their significance in ROC curves (AUC > 0.889). However, in our study we detected 33 metabolites and only one metabolite (leucine) overlapped in both studies. Although we identified 1 differential metabolite (acetylcarnitine) in CMT1A cohort, in the predictive model of CMT1A all metabolites showed poor accuracy for predicting the disease (AUC < 0.73) (Figure 3).

Due to CMT heterogeneity and the slow rate of progression, sensitive outcome measures and biomarkers are challenging to develop. Therefore, an association between potential biomarkers and disease severity is beneficial. Recently, several publications demonstrated a correlation between neurofilament light chain concentration in plasma and CMT disease severity (11, 12, 31, 32). In our study, we aimed to screen for potential metabolite biomarkers in varying severity degrees of CMT. Three candidate biomarkers (tyrosine, acetylcarnitine, proline) showed significant differences in pairwise analysis in mild versus severe and moderate versus severe CMT patient groups (Tables 5 and 6). However, we could not find significant changes in metabolites between all severity groups or with controls. Moreover, we looked for associations between CTM severity (CMTNSv2) and plasma metabolite levels in 66 CMT patients. Our study detected a poor correlation of all our measured metabolites (Supplementary Figure 2). In a previously published cohort (*n* = 42) by Soldevilla et al. (10)correlation analysis between metabolite levels and severity of the disease in CMT1A patients detected 5 metabolites (urobilinogen, glumatyl-serine, sphingosine-1-phosphate, palmitic amide, leucine) with good correlation (Spearman coefficient > 0.629). However, despite a larger cohort and more genetic CMT types, we could not replicate these results for leucine, the only overlapping metabolite present in our dataset. Therefore, more data and longitudinal evaluation are needed to establish whether metabolite markers can be used to monitor disease progression.

## Conclusion

Our study provides information about plasma metabolite levels in CMT patients. We have identified that CMT patients have significantly higher levels of acetylcarnitine and decreased glycine levels compared to controls. In addition, the CMTX1 subgroup has decreased valine levels compared to controls. Despite significant differences, our predictive models suggest no good predictive power of the detected serum metabolites for any CMT group. Furthermore, we found no associations of these metabolites with CMT severity. Consequently, the metabolites mentioned above might be unspecific biomarkers of neuropathy, however, longitudinal assessment is needed to evaluate metabolite marker capabilities.

### Limitations of the study

We provided data from a relatively large CMT cohort in the present study. However, the sample size between genetic subtypes varied. Another limitation was the small sample size in the severe CMT group compared to mild and moderate CMT. It should be noted that in the correlation analysis between detected metabolites and disease severity, we did not evaluate the association with the age of the patients. In this study, we focused on the metabolite set used in clinical diagnostics, e.g., amino acids and acylcarnitines. However, it is limited. Including other metabolites in our analysis or performing untargeted analysis could allow us to identify biomarkers with better performance.

## Data Availability

The raw data supporting the conclusions of this article will be made available by the authors, without undue reservation.

## Footnotes

^1^ www.metaboanalyst.ca
