# Supplementary material for: Metabolomics insights into Charcot–Marie–Tooth disease: toward biomarker discovery
Source: Front Neurol. 2025 May 19;16:1543547. doi: 10.3389/fneur.2025.1543547 (PMC12127190; doi:10.3389/fneur.2025.1543547)
Supplement: Supplementary file 1 [file Image_1.pdf]

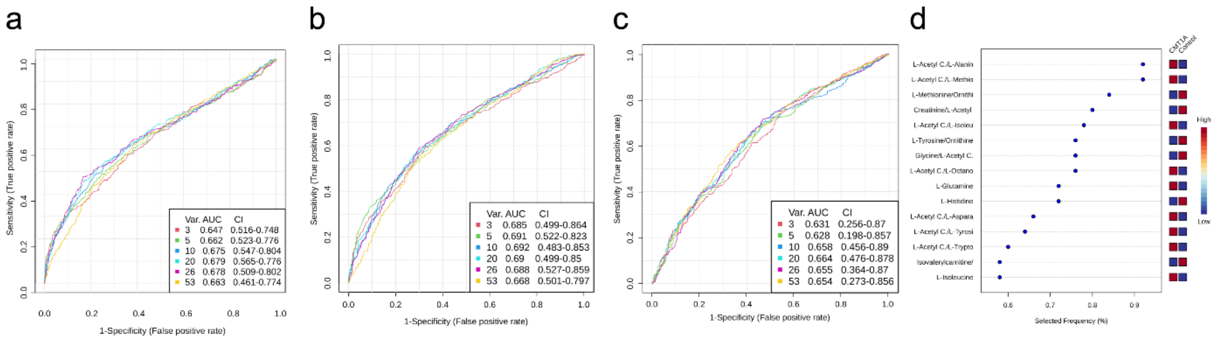

Supplementary figure 1. Comparison of classification models for CMT with different numbers of variables (right bottom box) using linear support vector machine algorithm for classification and RandomForest as ranking method for a. toral CMT, b. CMT1A, and c. CMTX1. d. Feature importance for classification of CMT1A.
