# Supplementary material for: Metabolomics insights into Charcot–Marie–Tooth disease: toward biomarker discovery
Source: Front Neurol. 2025 May 19;16:1543547. doi: 10.3389/fneur.2025.1543547 (PMC12127190; doi:10.3389/fneur.2025.1543547)
Supplement: Supplementary file 3 [file Table_1.pdf]

Metabolites	**4-Hydroxyproline**
	**Butyrylcarnitine**
	**Carnitine**
	**Citrulline**
	**Creatinine**
	**Glycine**
	**Isovalerylcarnitine**
	**Kynurenine**
	**L-Acetylcarnitine**
	**L-Alanine**
	**L-Arginine**
	**L-Asparagine**
	**L-Aspartic Acid**
	**L-Cystine**
	**L-Glutamic Acid**
	**L-Glutamine**
	**L-Histidine**
	**L-Leucine**
	**L-Isoleucine**
	**L-Lysine**
	**L-Methionine**
	**L-Octanoylcarnitine**
	**L-Phenylalanine**
	**L-Proline**
	**L-Serine**
	**L-Threonine**
	**L-Tryptophan**
	**L-Tyrosine**
	**L-Valine**
	**Methylhistidine**
	**Ornithine**
	**Propionylcarnitine**
	**Taurine**

Supplementary table 1. Plasma metabolites.
