# Supplementary material for: Metabolomics insights into Charcot–Marie–Tooth disease: toward biomarker discovery
Source: Front Neurol. 2025 May 19;16:1543547. doi: 10.3389/fneur.2025.1543547 (PMC12127190; doi:10.3389/fneur.2025.1543547)
Supplement: Supplementary file 2 [file Image_2.pdf]

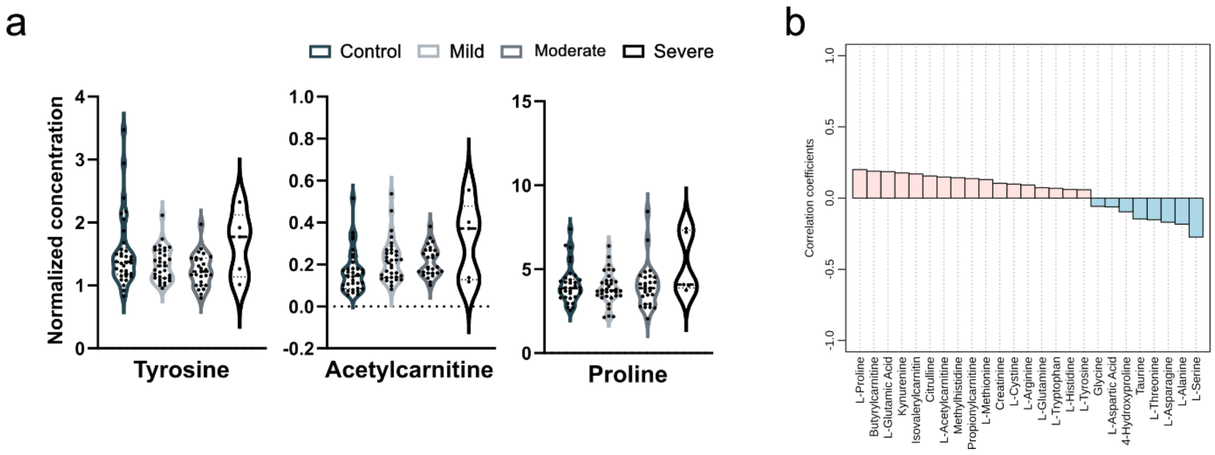

Supplementary figure 2a. Correlation of metabolites to severity scores in CMT. b. Concentrations of metabolites with the highest correlation score and the top-hit ratio with controls as a reference. Data was normalized to the mean of its corresponding sample, there is no significant difference between the groups (two-way ANOVA with Turkey's multiple comparison test).
